# Muscle Oxygen Delivery in the Forearm and in the *Vastus Lateralis* Muscles in Response to Resistance Exercise: A Comparison Between Nepalese Porters and Italian Trekkers

**DOI:** 10.3389/fphys.2020.607616

**Published:** 2020-11-10

**Authors:** Vittore Verratti, Danilo Bondi, Gabriele Mulliri, Giovanna Ghiani, Antonio Crisafulli, Tiziana Pietrangelo, Maria Erika Marinozzi, Paolo Cerretelli

**Affiliations:** ^1^Department of Psychological, Health and Territorial Sciences, University “G. d’Annunzio” of Chieti-Pescara, Chieti, Italy; ^2^Department of Neuroscience, Imaging and Clinical Sciences, University “G. d’Annunzio” of Chieti-Pescara, Chieti, Italy; ^3^Department of Medical Sciences and Public Health, University of Cagliari, Cagliari, Italy; ^4^Faculty of Veterinary Medicine, University of Teramo, Teramo, Italy; ^5^Institute of Bioimaging and Molecular Physiology, National Research Council of Italy, Segrate, Italy

**Keywords:** resistance exercise, hemodynamic response, Himalayas, near-infrared spectroscopy, hypobaric hypoxia

## Abstract

Altitude ascending represents an intriguing experimental model reproducing physiological and pathophysiological conditions sharing hypoxemia as the denominator. The aim of the present study was to investigate fractional oxygen extraction and blood dynamics in response to hypobaric hypoxia and to acute resistance exercises, taking into account several factors including different ethnic origin and muscle groups. As part of the “Kanchenjunga Exploration & Physiology” project, six Italian trekkers and six Nepalese porters took part in a high altitude trek in the Himalayas. The measurements were carried out at low (1,450 m) and high altitude (HA; 4,780 m). Near-infrared spectroscopy (NIRS)-derived parameters, i.e., Tot-Hb and tissue saturation index (TSI), were gathered at rest and after bouts of 3-min resistive exercise, both in the quadriceps and in the forearm muscles. TSI decreased with altitude, particularly in forearm muscles (from 66.9 to 57.3%), whereas the decrement was less in the quadriceps (from 62.5 to 57.2%); Nepalese porters were characterized by greater values in thigh TSI than Italian trekkers. Tot-Hb was increased after exercise. At altitude, such increase appeared to be higher in the quadriceps. This effect might be a consequence of the long-term adaptive memory due to the frequent exposures to altitude. Although speculative, we suggest a long-term adaptation of the Nepalese porters due to improved oxygenation of muscles frequently undergoing hypoxic exercise. Muscle structure, individual factors, and altitude exposure time should be taken into account to move on the knowledge of oxygen delivery and utilization at altitude.

## Introduction

Traveling at high altitude (HA) is nowadays very popular, but it requires medical advice in respect to altitude tolerance and acclimatization (Schommer and Bärtsch, 2011). In addition, altitude training has been widely investigated for its effects of environmental stressor on body homeostasis (Tam et al., 2016; Grocott et al., 2019). Common traveling conditions with Caucasian tourists along with local porters may have practical implications. For example, it may help in unraveling differences between native lowlanders and highlanders in response to hypobaric hypoxia (Magliulo et al., 2020), thereby shedding light on the physiological adaptation to living permanently or working occasionally at altitude. Moreover, high altitude hypoxia represents an intriguing experimental model to reproduce physiological and pathophysiological conditions commonly present at low altitude (LA), sharing hypoxemia as the common denominator (Di Giulio et al., 2003; Verratti et al., 2009; Sarkar et al., 2017): a classic example of long-life scientific interest is periodic breathing (Douglas, 1910). With regard to exercise physiology, most of the plans for altitude training were based on oxygen delivery and supply adaptations, with a faster response of O_2_ muscular uptake (Doria et al., 2011), an enhanced vascular endothelial growth factor (Vogt et al., 2001), along with a detrimental effect on muscle tissue over the period of typical Himalayan expeditions (Vogt and Hoppeler, 2010).

Oxygen delivery depends on cardiac output, arterial saturation and partial pressure of oxygen, and hemoglobin concentration. Oxygen supply is determined by oxygen delivery and carrying capacity, macro- and micro-vascular architecture, and blood flow dynamics. Direct oxygen consumption measurement and arteriovenous oxygen difference reflect the whole-body and local oxygen utilization. Studying local oxygen dynamics in field studies requires to use portable devices, along with feasible and non-invasive methods. Concerning the topic of field measurement, peripheral oxygenation and blood flow can be measured with the non-invasive technology of near-infrared spectroscopy (NIRS), based on the assumption of light-absorbing chromophores in skeletal muscle tissue, i.e., mainly hemoglobin (Hb) and myoglobin (Mb); thus, NIRS reflects the presence of heme in small vessels (<1 mm diameter; Barstow, 2019). Moreover, NIRS can be applied to different muscle groups, thereby allowing to discern regional adaptations, both in Lab settings (Volianitis et al., 2003) and during sport-specific exercise (Hesford et al., 2013), but no study has addressed the differences among muscle groups in hypobaric hypoxic field conditions.

Taking into account the limitations of the method, NIRS can provide valuable insight into the regional blood flow, skeletal muscle O_2_ consumption, fractional O_2_ extraction, and oxidative metabolic thresholds, during exercise and other conditions (Grassi and Quaresima, 2016). In this regard, many studies have addressed the topic of hypoxia, in terms of training (Fryer et al., 2019), acute (Peltonen et al., 2009), or chronic (Cheung et al., 2014) exposure. However, to the best of our knowledge, few studies if any, have investigated oxygen delivery and utilization in response to hypoxia comparing altitude (e.g., porters) to non-altitude workers.

Despite evidence of physiological differences among ethnic groups (Wu and Kayser, 2006), little is known about the differences in response to hypoxic conditioning. In this regard, Feeback et al. (2017) investigated arterial saturation and cerebral oxygenation in response to exercise hypoxia comparing African-American and Caucasian males, highlighting a differential response between the two ethnic groups. Simonson et al. (2015) highlighted the advantage of Tibetans in exerting higher exercise capacity at altitude to be supported by enhanced muscle O2 transport capacity. Thus, considering the adaptations in terms of chronic exposure and predisposition to altitude tolerance, it can be speculated that altitude populations or altitude workers may be different in blood flow and oxygen delivery and utilization in respect to lowlanders or non-altitude workers. We, therefore, hypothesized that Nepalese porters showed different adaptations as compared to Italian trekkers, due to a lesser impairment in oxygen delivery, supply and utilization, and in hemodynamic response to a middle-term altitude hypoxia exposure. We also hypothesized these adaptive differences to be present both at rest and in response to a resistance exercise at altitude. Thus, with the present study, we aimed to investigate the physiological adaptations in Nepalese porters vs. Italian trekkers during an altitude trek, considering different muscle groups, in response to strength exercise vs. rest.

## Materials and Methods

### Design and Participants

The research project “Kanchenjunga Exploration & Physiology” was a subset of “Environmentally-modulated metabolic adaptation to hypoxia in altitude natives and sea-level dwellers: from integrative to molecular (proteomics, epigenetics, and ROS) level” approved by the Ethical Review Board of the Nepal Health Research Council (NHRC). All study procedures were performed in accordance with the ethical standards of the 1964 Helsinki declaration and its later amendments or comparable ethical standards. Written informed consent was obtained from all participants.

The experimental subjects completed a combined circuit of 300 km length (south and north base camps), covering a daily walk average of 6 h, for a total of 110 h, along a demanding route with ascent and descent tracts, covering a total of over 16,000 m of vertical displacement in the Himalayan mountain range of eastern Nepal, at the border with Sikkim (India). The project investigated adaptive physiological responses to trekking throughout low (500–2,000 m), moderate (2,000–3,000 m), and high (3,000–5,500 m) altitudes (Schommer and Bärtsch, 2011), by two experimental groups composed, respectively by six healthy Caucasian lowlanders and six healthy Nepalese porters (see Table 1, for anthropometric characteristics). The Caucasian trekkers’ abode normally was at sea level and some of them reported previous high altitude experiences, although no one in the last 3 years. The Nepalese trekkers habitually live at LA and reported frequent exposure to high altitude, with a working experience of 2–5 similar expeditions per year in the last 3 years. The expedition was continuously supervised by an expert medical doctor; neither participants suffered from Acute Mountain Sickness during the trek, nor they reported any cardiovascular or respiratory disease. The average sleep duration was approximately 6–7 h for all participants. The Caucasian participants only took one acetazolamide pill of 250 mg daily, at 6 PM. during the 2 days between the acclimatization day and the stay at the highest altitude point of the expedition.

**Table 1:** Descriptive characteristics of participants and oxygen saturation measured in the same days of NIRS testing; Blood pressure (BP) is expressed as SystolicBP/DiastolicBP; group values are expressed as mean ± SD.

	Ethnicity	Sex	Age (years)	BMI (Kg/m^2^)	Basal BP (mmHg)	SpO_2_-LA (%)	SpO_2_-HA (%)
It1	Italian	Female	36	25.07	117/76	99	84
It2	Italian	Male	63	28.91	133/83	97	84
It3	Italian	Male	59	21.91	139/87	98	80
It4	Italian	Male	25	24.31	126/68	99	85
It5	Italian	Male	32	24.14	124/67	98	89
It6	Italian	Male	48	30.54	136/82	97	92
Italian Group			44 ± 15	25.81 ± 3.25	129 ± 8/77 ± 8	98 ± 1	86 ± 4
Ne1	Nepalese	Male	26	26.49	128/87	96	85
Ne2	Nepalese	Male	18	17.51	112/62	99	90
Ne3	Nepalese	Male	39	22.99	143/92	96	88
Ne4	Nepalese	Male	40	28.83	131/89	96	85
Ne5	Nepalese	Male	30	29.41	127/93	95	82
Ne6	Nepalese	Male	29	20.94	130/93	96	82
Nepalese group			30 ± 8	24.36 ± 4.70	129 ± 10/86 ± 12	96 ± 1	85 ± 3

BMI, body mass index; BP, blood pressure; SpO2, oxygen saturation; LA, low altitude; and HA, high altitude.

**Figure 1:**
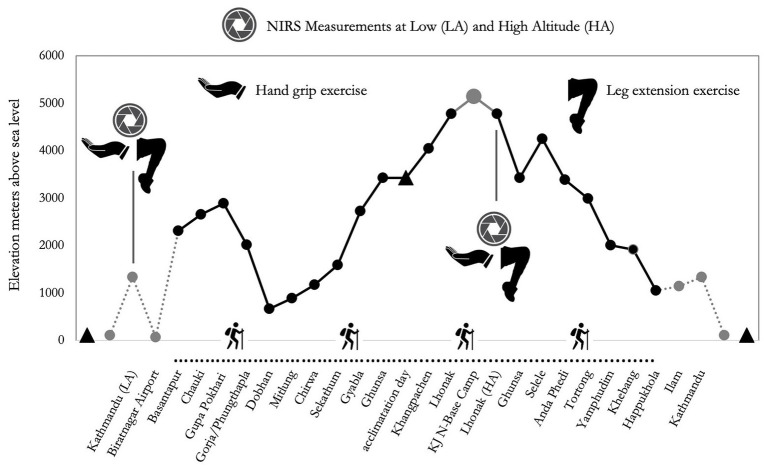
Altimetric plan of the “Kanchenjunga Exploration & Physiology” project. Measurements refer to near-infrared spectroscopy (NIRS) assessment, both on forearm (handgrip task) and anterior thigh (leg extension task).

From Kathmandu, the subjects were transferred to Biratnagar by plane to initiate the active phase of the expedition. From Biratnagar, participants reached Basantapur, a village at 2,300 m altitude, by an off-road vehicle. They then trekked to Lhonak at 4,780 in 12 days and on the 13th day reached the North Camp of Kanchenjunga. The control measurement at LA was carried out at Kathmandu (1,450 m), while those at HA were performed at Lhonak (4,780 m; see Figure 1). Considering the two groups planned the identical distance, and the groups trekked almost together, we analyzed the data of the wearable system of one Nepalese porter only to inform about the exercise load during the trek. Even though distance and difference in altitude were identical for the two groups, the workload was changing, reflecting the typical situation of modern altitude traveling: in fact, Caucasians carried light loads (up to 10 kg), whereas Nepalese carried heavier loads (up to 30 kg), along the whole route.

### Measurements

Trekking load was assessed with the wearable device Zephyr™ BioHarness 3 (Medtronic, United States), basing on accelerometry. Raw data were then analyzed by the software Omni Sense 5.0 (Medtronic, United States). Vector magnitude units (VMUs) were calculated as $\sqrt{x^{2}+y^{2}+z^{2}}$ where *x*, *y*, and *z* are the averages of the three axial acceleration magnitudes over the previous 1 s, sampled at 100 Hz. Axial accelerometer output was band-pass filtered, to remove non-human artifacts and gravity. Results are reported as gravity units, ranging from 0 to 16.

Measures by NIRS were performed in the quadriceps and forearm muscles. These were chosen mainly to include two different muscle groups, one directly involved in the walking effort, whereas the other was not. In addition, we chose *vastus lateralis* and anterior forearm muscles considering all the following tips: to limit the problem of adipose tissue contamination to NIRS signal, the lower thickness of those body parts in our participants (Barstow, 2019); because of the low presence of dark hair; because of the high simplicity to repeat the test; because largely used in NIRS assessments in previous studies (Jones et al., 2016); and because easy to set in respect to the bandages and logistics. To be specific, the NIRS probe was placed: (1) on the skin of the dominant forearm, over the middle part of the anterior compartment, and (2) on the skin of the dominant thigh, over the lower third of the *vastus lateralis* muscle. Both for forearm and thigh, an elastic dark bandage and an additional non-elastic dark bandage were applied over the probes.

After the positioning of the probes and the explanation of the exercise, without any warm-up, NIRS-derived parameters were collected for 3 min at rest; subsequently, during 3 min of exercise consisting in 18 concentric and eccentric contractions (5 s + 5 s) by the forearm muscles; and finally, 3 min of further recording was applied during recovery. Hereinafter, the 9 min of the protocol will be defined as stages. Then, after the re-positioning of the probe, the same procedure was repeated for the quadriceps. An operator paced the time. For forearm exercise, participants were required to grip and release a soft ball. For the quadriceps exercise, participants were required to extend and release the knee joint against an elastic training band, held by the same operator. Before testing, participants were allowed to attempt both kinds of exercise. This allowed them to get used to the instrumentation and to adapt their strength to the subsequent strain. For both forearm and thigh, the participants were asked to provide a progressive maximal effort and active release. To be specific, they were required to increase progressively the effort in 5 s to reach the maximal effort with a concentric contraction, and immediately release with an eccentric contraction during the following 5 s, back to resting condition, and so forth for 18 reps. The operator checked the real-time reactivity of parameters by the software, and continuously supported the participants for maintaining the exercise intensity and the pace. All tests were conducted in the late afternoon, after some hours of rest from daily trek. To ensure the exact re-positioning in different measurements, probes were placed onto the middle point of the forearm, and the one-third of the distance from the upper margin of the patella to the anterior superior iliac spine.

Near-infrared spectroscopy was used to assess the concentration changes of oxy-, deoxy- and total-hemoglobin (Tot-Hb), as measures of local vascular and metabolic response to muscle action, with a spatially resolved spectrometer specifically set to measure oxygenation in muscle tissue (PortaMon device and Oxysoft software, Artinis Medical Systems, Netherlands). PortaMon is a compact and lightweight device (75 g), containing three pairs of LEDs (at distances of 30, 35, and 40 mm from the silicon photodiode sensor) that uses the spatially resolved spectroscopy and the modified Beer-Lambert law (due to scattering and absorption in the tissue) to calculate the absolute concentration of chromophores. Multidistance continuous wave technique, with two different wavelengths (850 and 760 nm), and a sample rate of 10 Hz was used. Operating temperature requirements (10–35°C) were met. The usefulness of this device in measuring at rest and during exercise has already been reported (McManus et al., 2018). From the measures of oxy-Hb (O_2_Hb) and deoxy-Hb (HHb), we calculated to-Hb (O_2_Hb + HHb), diff-Hb (O_2_Hb − HHb), and tissue saturation index (TSI) as the percentage of oxygenated hemoglobin detected: TSI = (O_2_Hb) ÷ (O_2_Hb + HHb) × 100. Tot-Hb and TSI are indexes directly linked to local blood flow and oxygen consumption. To be noted that, throughout the text, we used Hb instead of (Hb + Mb); indeed, as suggested, it can be assumed that signal in mainly related to Hb, rather than Mb (Barstow, 2019). NIRS probe was posed parallel to the muscle, with a soft elastic bandage and two anelastic dark bandages to cover from ambient light. We calculated the minute-by-minute average of each parameter.

Peripheral oxygen saturation (SpO_2_) was measured in the same days of NIRS testing, using a pulse oximeter (APN-100, Contec Medical Systems Co. Ltd., China); values were considered allowing several seconds to detect a pulse and waiting for a stable value. The device measured in a range of 70–100% with an accuracy of 2%. The positioning of the rubber finger probe was done carefully, after cleaning and drying fingers (WHO, 2011), and the tests were performed in duplicate.

### Statistics

The statistical analysis and plots were carried out using R-based open source software Jamovi Version 1.2.5.0 (retrieved from https://www.jamovi.org) using GAMLj and Flexplot modules. Assumption check consisted of the Shapiro-Wilk test for normality of distributions, the Levene’s test for homogeneity of residual variances, and the Kolmogorov-Smirnov test for normality of residuals. Considering the results of the assumption check, the TSI values were undergone to log_10_ transformation while Tot-Hb values to square root transformation (even though with this transformation some missing points occurred, due to original negative values), then General Linear Mixed Model (GLMM) with Restricted Maximum Likelihood (REML) estimation method was used (Armstrong, 2017). Stage (1st, 2nd, 3rd, 7th, 8th, and 9th min), altitude (LA vs. HA), ethnicity (Nepalese vs. Italians), and muscle (quadriceps vs. forearm) were set as fixed factors and participants as a random factor. Two-way interactions were considered. Fit measures [Akaike Information Criterion (AIC) and Bayesian Information Criterion (BIC)] were reported, as well as marginal *R*^2^ (proportion of variance explained by the fixed factors only), and conditional *R*^2^ (proportion of variance explained by the fixed and random factors) and Likelihood Ratio Test (LRT) for the random effect. The effect size of fixed factors was calculated and reported as Cohen’s *f*^2^ (Selya et al., 2012).

## Results

The trek was conducted with average gravity units of 0.18 in the 1st day of monitoring (3rd day of trek, at moderate altitude, and 16.2 km of distance covered), and 0.15 in the 2nd day (16th day of trek, at high altitude, and 7.8 km of distance covered). To be noted that 0.2 corresponds to a normal walk. The results we obtained averaged also the resting phase during the daily trek, and the total workload consisted also of the carrying. Two Nepalese carried 10–20 kg daily, the others four Nepalese 25–30 kg daily. The Italians carried 2–10 kg daily.

As expected, oxygen saturation decreased with altitude in all participants, ranging from 80 to 92% at high altitude; all participants showed values in the normal physiological range at LA (see Table 1). Model fit measures suggested that TSI had a better fit of the current analyses compared to Tot-Hb. In both cases, individuality significantly affected the analyses, especially for Tot-Hb (see LRT values on Table 2).

**Table 2:** General Linear Mixed Model (GLMM) statistics of NIRS parameters; stage, ethnicity, muscle and altitude: fixed factors; participants: random factor.

	TSI	Tot-Hb
AIC	−1,082	603
BIC	−572	786
*R*^2^ marginal	0.435	0.414
*R*^2^ conditional	0.468	0.575
Random LRT	*p* = 0.022	*p* < 0.001
Stage	*p* = 0.898	*p* < 0.001
Ethnicity	*p* = 0.396	*p* = 0.485
Muscle	*p* < 0.001	*p* = 0.588
Altitude	*p* < 0.001	*p* = 0.710
Stage × ethnicity	*p* = 0.997	*p* = 0.896
Stage × muscle	*p* = 0.753	*p* = 0.927
Muscle × ethnicity	*p* < 0.001	*p* = 0.234
Stage × altitude	*p* = 0.992	*p* = 0.850
Altitude × ethnicity	*p* = 0.117	*p* = 0.918
Muscle × altitude	*p* < 0.001	*p* = 0.008

The lesser are fit measures [Akaike Information Criterion (AIC) and Bayesian Information Criterion (BIC)], the better is the model. Stage (1st, 2nd, 3rd, 7th, 8th, and 9th min), altitude (LA vs. HA), ethnicity (Nepalese vs. Italians), and muscle (quadriceps vs. forearm) were set as fixed factors and participants as a random factor. Two-way interactions were considered and reported. TSI, tissue saturation index; Tot-Hb, total hemoglobin; AIC, akaike information criterion; BIC, bayesian information criterion; LRT, likelihood ratio test.

**Figure 2:**
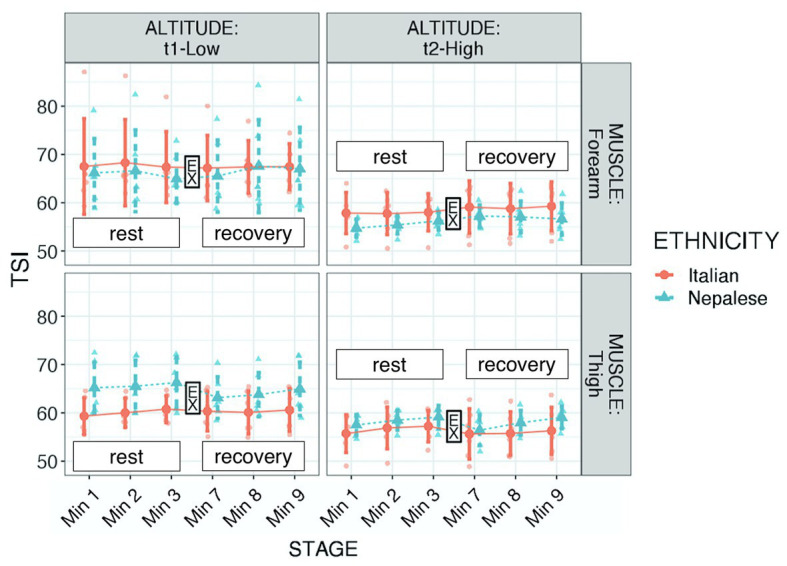
Tissue saturation index (TSI) of participants during “Kanchenjunga Exploration & Physiology” project. Measurements were done with a NIRS device on forearm (**upper panels**) and anterior thigh (**lower panels**), 3 min before (rest) and 3 min after (recovery) a 3-min-lasting exercise. EX represents the 3-min time spot of resistance exercise, constituted by 18 submaximal contractions. Both groups (Italians and Nepalese) were measured at low (1,450 m, **left panels**) and high (4,750 m, **right panels**) altitude. Data represent single values with mean ± SD, averaged by 1-min recording.

Regarding TSI, significant differences were found in respect to muscle (higher values in forearm: *p* < 0.001, *f*^2^ = 0.158), altitude (higher values at LA: *p* < 0.001, *f*^2^ = 0.695), ethnicity × muscle (Nepalese had higher values in thigh: *p* < 0.001, *f*^2^ = 0.156), and muscle × altitude (higher decrease in forearm with altitude: *p* < 0.001, *f*^2^ = 0.833), and a tendency for ethnicity × altitude (lesser decrease in Italians with altitude: *p* = 0.117, *f*^2^ = 0.776; see Table 2; Figure 2). Mean parameters before and after exercise are reported in Table 3.

**Table 3:** Tissue saturation index (TSI) values, obtained from NIRS measurement, before and after the exercise, clustered by ethnic group, muscle group, and altitude.

	Altitude	Forearm muscle		*Vastus lateralis*	
		Before Ex	After Ex	Before Ex	After Ex
Italians trekkers	Low	67.8 ± 8.7	67.4 ± 5.6	60.0 ± 3.2	60.4 ± 4.3
	High	57.9 ± 4.0	59.1 ± 5.2	56.6 ± 3.7	55.9 ± 4.8
Nepalese porters	Low	65.9 ± 6.5	66.7 ± 8.5	65.7 ± 5.3	64.0 ± 4.7
	High	55.5 ± 1.8	57.0 ± 2.9	58.4 ± 2.1	57.8 ± 2.7

Data are percent values and are reported as mean ± SD. Ex: Exercise; TSI = (O_2_Hb) ÷ (O_2_Hb + HHb) × 100.

**Figure 3:**
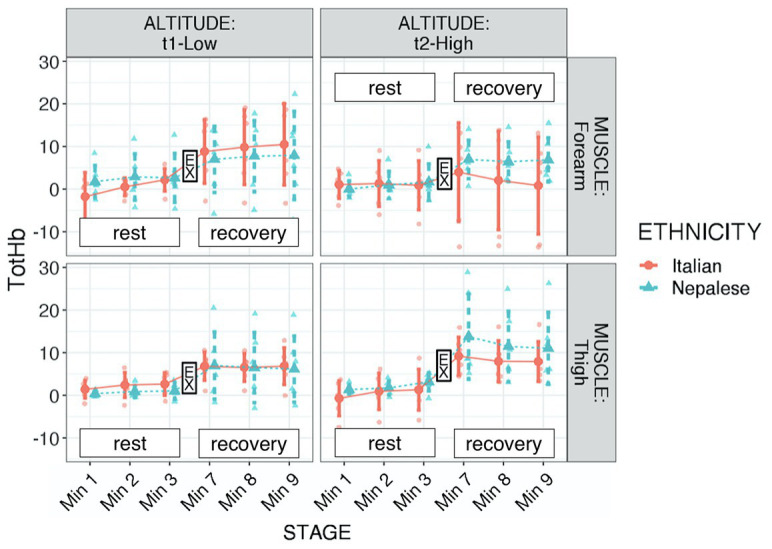
Total hemoglobin (tot-HB) of participants during “Kanchenjunga Exploration & Physiology” project. Measurements were done with a NIRS device on forearm (**upper panels**) and anterior thigh (**lower panels**), 3 min before (rest) and 3 min after (recovery) a 3-min-lasting exercise. Exercise (EX) represents the 3-min time spot of resistance exercise, constituted by 18 submaximal contractions. Both groups (Italians and Nepalese) were measured at low (1,450 m, **left panels**) and high (4,750 m, **right panels**) altitude. Data represent single values with mean ± SD, averaged by 1-min recording.

Regarding tot-Hb, significant differences were found in respect to stage (increment after exercise: *p* < 0.001, *f*^2^ = 0.913) and muscle × altitude (higher increase in thigh with altitude: *p* = 0.008, *f*^2^ = 0.052; see Table 2; Figure 3). Considering the behavior of this parameter, we additionally ran the analysis setting the average at the 3rd-min-before exercise and the 3rd-min-after exercise, instead of six stages. Those values underwent the same assumption check and consequently a square root transformation was applied. The robust evidence was the strong increment after exercise (*p* < 0.001, *f*^2^ = 1.045). Although only descriptive, as shown in Figure 3, it seemed that Nepalese could achieve a more consistent blood flow increase in the exercised muscle.

## Discussion

From an epistemological point of view, the MEDLINE database revealed a systematic increase in the adaptive responses of humans to high altitude since the second half of the 19th century (Donaldson, 1888). This interest had been cultivated with a certain constancy in the following years, but only since the ‘50s occurred an exponential growth in quantity and quality of the related articles (Margaria, 1951; Swan, 1953; Cerretelli and Margaria, 1961). This phenomenon was strictly connected to the desire of generations of climbers, who followed dreams of glory and aimed to conquer the 14 mountains above 8,000 m (Messner, 1999). This opened an intriguing door for those scholars interested in studying extreme adaptations (West, 1990; Grocott et al., 2009). In recent years, a 3-fold alliance has been appearing, linking climbers and scientists with local workers (porters and guides), who offered skills and knowledge to realize expeditions with mountaineering and scientific purposes.

The altitude increase generates a simultaneous and roughly linear reduction in atmospheric pressure and inspired oxygen pressure (Peacock, 1998). Respiration, oxygen transport, and alveolar-arterial oxygen-tension gradient regulate the oxygen supply to the new metabolic needs that the high altitude presents cardiac output and hemoglobin concentration. Oxygen delivery and supply are the crucial topic in hypobaric hypoxic adaptations (West, 1990; O’Brien et al., 2018).

With the present study, we investigated fractional oxygen extraction and reactive hyperemia during an altitude expedition, through the NIRS method. We aimed to compare two common groups of altitude travelers (Nepalese vs. Caucasian) and two diverse muscle groups (forearm and anterior thigh) at low and high altitudes, before and in response to a submaximal exercise. The originality of the work consisted in the possibility to include NIRS equipment within the luggage during a high altitude trek, allowing of conducting field-based measurements in two diverse ethnic groups.

First, the decrease of peripheral oxygen saturation revealed through the pulse oximeter in both groups, proved the known adaptations of altitude sojourn in our model. In addition, the duration of exercise (3 min with 18 submaximal repetitions) allowed observing a marked and sustained hemodynamic response. With this regard, the NIRS method optimally fitted our model, unveiling valuable insights into the regional blood dynamics and muscle oxygenation. Between the two parameters, we evaluated (TSI and Tot-Hb), TSI fitted better the current analysis; both were significantly affected by individuality, Tot-Hb to a greater extent.

As expected, at altitude, TSI decreased. This reduction was particularly evident in the forearm muscle. The reason for this difference in muscle groups may have two reasons: (1) both muscle groups reached low values of saturation, thus starting from higher values, the forearm muscle may have been reached that critical threshold. On average, TSI moved from 66.9 to 57.3% in the forearm and from 62.5 to 57.2% in the thigh. Although speculative, we may indeed interpret 57% as a critical threshold, at least in our findings and (2) a different interpretation lies on middle-term adaptation: as discussed below, the exercise carried on a greater extent by lower limb muscles may have inhibited tissue desaturation in the thigh. We also found a difference between the ethnic groups: the Nepalese porters had greater values in thigh TSI than the Italian trekkers. This result may lie on long-term adaptive memory of porters, due to the frequent exposure to altitude load-carrying trek. Hence, the greater workload of lower limbs may lead the selective adaptation of these muscle groups (Tam et al., 2016) in altitude porters. Therefore, frequent exercise protocols at altitude may produce increases in local tissue oxygenation.

It seemed that Italians had a lesser reduction with altitude; this result may have been related to the lighter load carried by this group. Martin et al. (2009) showed that tissue oxygen saturation in the *vastus lateralis* decreases during maximal oxygen consumption to increase again during the recovery phase. The same authors revealed how the response pattern to different stages of the exercise protocol was similar at moderate and high altitude in respect to sea level, although rest values were lower at all stages in altitude. Furthermore, the exposure to extremely high altitudes had a protective effect during exercise, reducing the degree of de-saturation. In respect to the maximal aerobic exercise, in our model, tissue saturation was not influenced by the resistive exercise, opening the way to a prospective new study determining differences in response to hypoxic exercise in terms of exercise typology. Thus, high altitude may represent a stressor to the oxygen system, capable of entailing beneficial effects in oxygen delivery and utilization (Tam et al., 2016), in addition to muscular structural and functional changes (Doria et al., 2011). Prospectively, the specific definition of exercise loads should drive the plan of exercise protocols at altitude for stressing the local tissue oxygenation response. There were no changes in TSI after the submaximal exercise; this result agrees with the recent findings of Yoshiko et al. (2020), who reported a significant deoxygenation during hypoxic exercise, but not in the recovery.

Hemodynamic response to submaximal resistive exercise showed an increase in blood flow, as estimated by the Tot-Hb parameter. At altitude, it seemed that this increase was particularly high in the thigh rather than in the forearm muscle. In addition, even though our model did not allow the three-way statistical analysis, from Figure 3 it can be observed a difference between ethnic groups: Nepalese porters may have required a higher blood flow increment after exercise in altitude, both in the forearm and in the thigh muscle. The marked hemodynamic response to submaximal dynamic exercise, as found in the present study, might be further characterized in terms of hemodynamic reserves (Bassareo and Crisafulli, 2020): hypoxia has a role in muscle metaboreflex (Mulliri et al., 2019), and the dynamics of diverse muscle groups can be stressed to achieve further insight. This response might be the result of systemic advantage, achieved by heritable factors or due to long-term adaptation, representing a likely basis for a further adaptation in exercised muscles. Therefore, the adequate definition of blood flow response to isometric vs. dynamic exercise under hypoxic conditions is required to define the training methods.

Concerning ethnicity, it has been suggested that the physiological advantages of Andeans may be related to a greater efficiency in oxygen transfer and utilization, supported by a likely genetic adaptation, non-selective to oxygen-sensitive genes (Julian and Moore, 2019). On the Himalayan side, genetic and epigenetic bases of Sherpas were extensively studied in Xtreme Everest 2 project (Martin et al., 2013) whose major conclusion, among others, was that Peroxisome Proliferator Activated Receptor Alpha (PPARA) gene may entail the metabolic basis to permit a superior survival and performance at high altitude (Horscroft et al., 2017). Beyond ethnicity, Puthon et al. (2016) described how elite climbers can elicit advantages with respect to trained non-climber controls: these authors suggested that these advantages were the consequence of a lower hypoxic and hypercapnic ventilatory response rather than to an enhanced peripheral saturation. Wang et al. (2010) showed that 4-week hypoxic exercise training enhanced oxygen perfusion and utilization in exercising skeletal muscles. Thus, we may speculate about a likely long-term adaptation of the Nepalese porters to improved oxygenation in those muscles involved in hypoxic exercise (herein altitude trekking). At least in part, this adaptation may lie on the better capability to increase the regional blood flow. One plausible pathway possibly involved lies on the nitric oxide (NO) system, known to be a major contributor of the compensatory vasodilation in response to hypoxia (Joyner and Casey, 2014). Here, we suggest ethnic differences may lie in some non-NO synthase pathways (Lepore, 2000), excluding nutritional factors (Luiking et al., 2010). Extending pieces of evidence on genetic advantages of altitude populations, further studies may investigate possible genetic advantages of acute or middle-term response to hypobaric hypoxia. Such an investigation may address the intriguing world of potentially heritable epigenetic factors.

Individuality had a significant effect on both NIRS parameters, more marked for Tot-Hb. Thus, further studies on a larger sample size and more accurate control of individual factors (e.g., age, sex, metabolic diseases, frequency of altitude exposure, time since last exposure, recent injuries, cardiovascular measures, and muscle structure) should extend our results. Indeed, differences in age and carried loads between the two groups could have affected the results we obtained. Differences in intrinsic characteristics of the forearm and the thigh muscles may also have played a role: Yoshiko et al. (2020) recently reported some differences in the diverse muscles of *quadriceps* comparing deoxygenation during submaximal exercise in normoxic and hypoxic trials. Thus, it should be of interest to study the diverse behavior of muscles basing of morpho-functional characterization. Ethnic differences in the muscle structural and functional characteristics may affect the response to hypoxia and should deserve further investigation: despite evidence of muscular inborn characteristics of Himalayan population (Kayser et al., 1996), the high heterogeneity of Caucasian ethnic group does not allow to speculate about likely ethnic difference in muscle composition for affecting the current results.

### Limitations

Like other methods, NIRS investigates a small and relatively superficial portion of the muscle, thus those portions cannot be assumed as representative for the whole muscle. Several confounding variables, i.e., age difference, unequal sex distribution within the two groups, and the difference in the load carried, some participants having elevated blood pressure. The anthropometric and training status of participants may also have affected the results. The exercise protocol during the NIRS measurements were not quantitatively measured, due to the logistical difficulty to provide a real-time monitoring of dynamometer data in field conditions. The small sample size makes difficult to derive firm conclusions. Workload, medicine intake, diet, and sleep were not standardized between groups. However, these conditions were due given the nature of this work, i.e., an outdoor field-based study conducted in a harsh environment.

### Perspectives

The pieces of evidence of the present work further support investigations on this topic, designing outdoor field studies with the accurate external and internal workload, metabolic performance characterization, and follow-up assessment. Some additional measures of very thin vessels and some clustering based on likely different pattern of hemodynamic response during exercise (Dech et al., 2020) may add new levels of comprehension. Further studies may extend these results verifying whether the common principle of climbers “Climb High and sleep low” based on induced hypoxia (pre)conditioning for a safe rapid ascent to extreme altitudes (Burtscher and Koch, 2016) may lead the optimal response to exercise in terms of muscle oxygen saturation.

Intriguing perspectives lie on cross adaptation: the use of one stimulus (herein altitude hypoxia) to model the responses to a similar stimulus in other circumstances (e.g., clinics; Lee et al., 2019). Moreover, considering the differences we found between muscle groups, it remains to be investigated whether hypoxia modulates tissue cross-talk. Exercised muscles provide a means for systemic cross-talk, both from a molecular (Whitham et al., 2018) and a functional (Pietrangelo et al., 2019) basis. Thus, it should be of interest to verify the adaptation of muscle-related cross-talk in response to hypobaric hypoxia.

In conclusion, individual factors should be taken into account if oxygen delivery and utilization are addressed. Muscular morpho-functional characteristics may unveil further determinants of specific responses to hypoxia. The combined use of both saturation and blood flow indexes deserves further investigation to clarify the role of ethnicity, muscle group, and hypoxic condition in response to altitude physical exercise.

## Data Availability Statement

The raw data supporting the conclusions of this article will be made available by the authors, without undue reservation.

## Ethics Statement

The studies involving human participants were reviewed and approved by Ethical Review Board of the Nepal Health Research Council. The patients/participants provided their written informed consent to participate in this study.

## Author Contributions

VV, DB, GM, AC, and TP: conceptualization. VV, DB, GM, GG, AC, and TP: methodology. VV, DB, GM, GG, and AC: formal analysis. DB and MM: investigation. VV and AC: resources. DB and GM: data curation. VV, DB, AC, and TP: writing – original draft preparation. VV, DB, GM, GG, AC, TP, MM, and PC: writing – review and editing. DB: visualization. VV, AC, and PC: supervision. VV: project administration and funding acquisition. All authors contributed to the article and approved the submitted version.

### Conflict of Interest

The authors declare that the research was conducted in the absence of any commercial or financial relationships that could be construed as a potential conflict of interest.

## References

[ref1] Armstrong R. A. (2017). Recommendations for analysis of repeated-measures designs: testing and correcting for sphericity and use of MANOVA and mixed model analysis. Ophthalmic Physiol. Opt. 37, 585–593. 10.1111/opo.12399, PMID: 28726257

[ref2] Barstow T. J. (2019). Understanding near infrared spectroscopy and its application to skeletal muscle research. J. Appl. Physiol. 126, 1360–1376. 10.1152/japplphysiol.00166.2018, PMID: 30844336

[ref3] Bassareo P. P. Crisafulli A. (2020). Gender differences in hemodynamic regulation and cardiovascular adaptations to dynamic exercise. Curr. Cardiol. Rev. 16, 65–72. 10.2174/1573403X15666190321141856, PMID: 30907327 PMC7393595

[ref4] Burtscher M. Koch R. (2016). Effects of pre-acclimatization applying the “climb high and sleep low” maxim: an example of rapid but safe ascent to extreme altitude. J. Hum. Perf. Extrem. Environ. 12:2. 10.7771/2327-2937.1081

[ref5] Cerretelli P. Margaria R. (1961). Maximum oxygen consumption altitude. Int. Z. Angew. Physiol. 18, 460–464. 10.1007/bf00699458, PMID: 13692019

[ref6] Cheung S. S. Mutanen N. E. Karinen H. M. Koponen A. S. Kyröläinen H. Tikkanen H. O.. (2014). Ventilatory chemosensitivity, cerebral and muscle oxygenation, and total hemoglobin mass before and after a 72-day mt. Everest expedition. High Alt. Med. Biol. 15, 331–340. 10.1089/ham.2013.1153, PMID: 25211648

[ref8] Dech S. Bittmann F. Schaefer L. (2020). Behavior of oxygen saturation and blood filling in the venous capillary system of the biceps brachii muscle during a fatiguing isometric action. Eur. J. Transl. Myol. 30:8800. 10.4081/ejtm.2019.8800, PMID: 32499884 PMC7254419

[ref9] Di Giulio C. Bianchi G. Cacchio M. Macrì M. A. Ferrero G. Rapino C.. (2003). Carotid body HIF-1alpha, VEGF and NOS expression during aging and hypoxia. Adv. Exp. Med. Biol. 536, 603–610. 10.1007/978-1-4419-9280-2_76, PMID: 14635718

[ref10] Donaldson F. (1888). Further remarks on the circulatory changes at high altitude. Trans. Am. Climatol. Assoc. Meet. 5, 96–99. PMID: 21407369 PMC2526721

[ref11] Doria C. Toniolo L. Verratti V. Cancellara P. Pietrangelo T. Marconi V.. (2011). Improved VO2 uptake kinetics and shift in muscle fiber type in high-altitude trekkers. J. Appl. Physiol. 111, 1597–1605. 10.1152/japplphysiol.01439.2010, PMID: 21868681

[ref12] Douglas C. G. (1910). Periodic breathing at high altitudes. J. Physiol. 40, 454–471. 10.1113/jphysiol.1910.sp001382, PMID: 16993020 PMC1533692

[ref13] Feeback M. R. Seo Y. Dancy M. Glickman E. L. (2017). The effect of psychomotor performance, cerebral and arterial blood saturation between African-American and Caucasian males before, during and after normobaric hypoxic exercise. Int. J. Exerc. Sci. 10, 655–665. 28966706 10.70252/JPAE3034 PMC5609664

[ref14] Fryer S. Stone K. Dickson T. Wilhelmsen A. Cowen D. Faulkner J.. (2019). The effects of 4 weeks normobaric hypoxia training on microvascular responses in the forearm flexor. J. Sports Sci. 37, 1235–1241. 10.1080/02640414.2018.1554177, PMID: 30558476

[ref15] Grassi B. Quaresima V. (2016). Near-infrared spectroscopy and skeletal muscle oxidative function in vivo in health and disease: a review from an exercise physiology perspective. J. Biomed. Opt. 21:091313. 10.1117/1.JBO.21.9.091313, PMID: 27443955

[ref16] Grocott M. P. W. Levett D. Z. H. Ward S. A. (2019). Exercise physiology: exercise performance at altitude. Curr. Opin. Physiol. 10, 210–218. 10.1016/j.cophys.2019.06.008

[ref17] Grocott M. P. W. Martin D. S. Levett D. Z. H. McMorrow R. Windsor J. Montgomery H. E. (2009). Arterial blood gases and oxygen content in climbers on Mount Everest. N. Engl. J. Med. 360, 140–149. 10.1056/NEJMoa0801581, PMID: 19129527

[ref18] Hesford C. M. Laing S. Cooper C. E. (2013). Using portable NIRS to compare arm and leg muscle oxygenation during roller skiing in biathletes: a case study. Adv. Exp. Med. Biol. 789, 179–184. 10.1007/978-1-4614-7411-1_25, PMID: 23852493

[ref19] Horscroft J. A. Kotwica A. O. Laner V. West J. A. Hennis P. J. Levett D. Z. H.. (2017). Metabolic basis to Sherpa altitude adaptation. Proc. Natl. Acad. Sci. U. S. A. 114, 6382–6387. 10.1073/pnas.1700527114, PMID: 28533386 PMC5474778

[ref20] Jones S. Chiesa S. T. Chaturvedi N. Hughes A. D. (2016). Recent developments in near-infrared spectroscopy (NIRS) for the assessment of local skeletal muscle microvascular function and capacity to utilise oxygen. Artery Res. 16, 25–33. 10.1016/j.artres.2016.09.001, PMID: 27942271 PMC5134760

[ref21] Joyner M. J. Casey D. P. (2014). Muscle blood flow, hypoxia, and hypoperfusion. J. Appl. Physiol. 116, 852–857. 10.1152/japplphysiol.00620.2013, PMID: 23887898 PMC3972742

[ref22] Julian C. G. Moore L. G. (2019). Human genetic adaptation to high altitude: evidence from the Andes. Genes 10:150. 10.3390/genes10020150, PMID: 30781443 PMC6410003

[ref23] Kayser B. Hoppeler H. Desplanches D. Marconi C. Broers B. Cerretelli P. (1996). Muscle ultrastructure and biochemistry of lowland Tibetans. J. Appl. Physiol. 81, 419–425. 10.1152/jappl.1996.81.1.419, PMID: 8828694

[ref24] Lee B. J. Gibson O. R. Thake C. D. Tipton M. Hawley J. A. Cotter J. D. (2019). Editorial: cross adaptation and cross tolerance in human health and disease. Front. Physiol. 9:1827. 10.3389/fphys.2018.01827, PMID: 30670977 PMC6331449

[ref25] Lepore D. A. (2000). Nitric oxide synthase-independent generation of nitric oxide in muscle ischemia–reperfusion injury. Nitric Oxide 4, 541–545. 10.1006/niox.2000.0308, PMID: 11139361

[ref26] Luiking Y. C. Engelen M. P. K. J. Deutz N. E. P. (2010). Regulation of nitric oxide production in health and disease. Curr. Opin. Clin. Nutr. Metab. Care 13, 97–104. 10.1097/MCO.0b013e328332f99d, PMID: 19841582 PMC2953417

[ref27] Magliulo L. Bondi D. Pietrangelo T. Fulle S. Piccinelli R. Jandova T. (2020). Serum ferritin and vitamin D evaluation in response to high altitude comparing Italians trekkers vs Nepalese porters. Eur. J. Sport Sci. 1–21. 10.1080/17461391.2020.1792559 32627691

[ref28] Margaria R. (1951). Respiration at low barometric pressure. Minerva Med. 42, 1235–1242. PMID: 14919291

[ref29] Martin D. S. Gilbert-Kawai E. Levett D. Z. Mitchell K. Kumar B. C. R. Mythen M. G.. (2013). Xtreme Everest 2: unlocking the secrets of the Sherpa phenotype? Extrem. Physiol. Med. 2:30. 10.1186/2046-7648-2-30, PMID: 24229457 PMC3853703

[ref30] Martin D. S. Levett D. Z. H. Mythen M. Grocott M. P. W. Caudwell Xtreme Everest Research Group (2009). Changes in skeletal muscle oxygenation during exercise measured by near-infrared spectroscopy on ascent to altitude. Crit. Care 13:S7. 10.1186/cc8005, PMID: 19951391 PMC2786109

[ref31] McManus C. J. Collison J. Cooper C. E. (2018). Performance comparison of the MOXY and PortaMon near-infrared spectroscopy muscle oximeters at rest and during exercise. J. Biomed. Opt. 23, 1–14. 10.1117/1.JBO.23.1.015007, PMID: 29368457

[ref32] Messner R. (1999). All 14 eight-thousanders. Seattle, USA: Mountaineers Books.

[ref33] Mulliri G. Sainas G. Magnani S. Roberto S. Ghiani G. Mannoni M.. (2019). Effects of exercise in normobaric hypoxia on hemodynamics during muscle metaboreflex activation in normoxia. Eur. J. Appl. Physiol. 119, 1137–1148. 10.1007/s00421-019-04103-y, PMID: 30783735

[ref34] O’Brien K. A. Pollock R. D. Stroud M. Lambert R. J. Kumar A. Atkinson R. A.. (2018). Human physiological and metabolic responses to an attempted winter crossing of Antarctica: the effects of prolonged hypobaric hypoxia. Phys. Rep. 6:e13613. 10.14814/phy2.13613, PMID: 29521037 PMC5843758

[ref35] Peacock A. J. (1998). ABC of oxygen: oxygen at high altitude. BMJ 317, 1063–1066. 10.1136/bmj.317.7165.1063, PMID: 9774298 PMC1114067

[ref36] Peltonen J. E. Paterson D. H. Shoemaker J. K. Delorey D. S. Dumanoir G. R. Petrella R. J.. (2009). Cerebral and muscle deoxygenation, hypoxic ventilatory chemosensitivity and cerebrovascular responsiveness during incremental exercise. Respir. Physiol. Neurobiol. 169, 24–35. 10.1016/j.resp.2009.08.013, PMID: 19729079

[ref37] Pietrangelo T. Bondi D. Kinel E. Verratti V. (2019). The bottom-up rise strength transfer in elderly after endurance and resistance training: the BURST. Front. Physiol. 9:1944. 10.3389/fphys.2018.01944, PMID: 30692938 PMC6339983

[ref38] Puthon L. Bouzat P. Rupp T. Robach P. Favre-Juvin A. Verges S. (2016). Physiological characteristics of elite high-altitude climbers. Scand. J. Med. Sci. Sports 26, 1052–1059. 10.1111/sms.12547, PMID: 26314388

[ref39] Sarkar M. Niranjan N. Banyal P. (2017). Mechanisms of hypoxemia. Lung India 34, 47–60. 10.1016/s1078-5337(05)70087-3, PMID: 28144061 PMC5234199

[ref40] Schommer K. Bärtsch P. (2011). Basic medical advice for travelers to high altitudes. Dtsch. Arztebl. Int. 108:839. 10.3238/arztebl.2011.0839, PMID: 22238560 PMC3254048

[ref41] Selya A. S. Rose J. S. Dierker L. C. Hedeker D. Mermelstein R. J. (2012). A practical guide to calculating cohen’s f(2), a measure of local effect size, from PROC MIXED. Front. Psychol. 3:111. 10.3389/fpsyg.2012.00111, PMID: 22529829 PMC3328081

[ref42] Simonson T. S. Wei G. Wagner H. E. Wuren T. Qin G. Yan M.. (2015). Low haemoglobin concentration in Tibetan males is associated with greater high-altitude exercise capacity. J. Physiol. 593, 3207–3218. 10.1113/JP270518, PMID: 25988759 PMC4532538

[ref7] Swan C. (1953). Climbing Everest: some notes on the medical team. Br. Med. J. 1, 1329–1330. PMC2016561 13042245

[ref43] Tam E. Bruseghini P. Calabria E. Dal Sacco L. Doria C. Grassi B.. (2016). Gokyo Khumbu/Ama Dablam trek 2012: effects of physical training and high-altitude exposure on oxidative metabolism, muscle composition, and metabolic cost of walking in women. Eur. J. Appl. Physiol. 116, 129–144. 10.1007/s00421-015-3256-z, PMID: 26349745

[ref44] Verratti V. Giulio C. D. Bianchi G. Cacchio M. Petruccelli G. Artese L. (2009). “Neuroglobin in aging carotid bodies” in Arterial chemoreceptors: Advances in experimental medicine and biology. eds. Gonzalez C. Nurse C. A. Peers C. (Dordrecht: Springer Netherlands), 191–195. 10.1007/978-90-481-2259-2_22 19536481

[ref45] Vogt M. Hoppeler H. (2010). Is hypoxia training good for muscles and exercise performance? Prog. Cardiovasc. Dis. 52, 525–533. 10.1016/j.pcad.2010.02.013, PMID: 20417346

[ref46] Vogt M. Puntschart A. Geiser J. Zuleger C. Billeter R. Hoppeler H. (2001). Molecular adaptations in human skeletal muscle to endurance training under simulated hypoxic conditions. J. Appl. Physiol. 91, 173–182. 10.1152/jappl.2001.91.1.173, PMID: 11408428

[ref47] Volianitis S. Krustrup P. Dawson E. Secher N. H. (2003). Arm blood flow and oxygenation on the transition from arm to combined arm and leg exercise in humans. J. Physiol. 547, 641–648. 10.1113/jphysiol.2002.034496, PMID: 12562897 PMC2342644

[ref48] Wang J. -S. Wu M. -H. Mao T. -Y. Fu T. Hsu C. -C. (2010). Effects of normoxic and hypoxic exercise regimens on cardiac, muscular, and cerebral hemodynamics suppressed by severe hypoxia in humans. J. Appl. Physiol. 109, 219–229. 10.1152/japplphysiol.00138.2010, PMID: 20431021

[ref49] West J. B. (1990). Tolerance to severe hypoxia: lessons from Mt. Everest. Acta Anaesthesiol. Scand. 34, 18–23. 10.1111/j.1399-6576.1990.tb03216.x, PMID: 2127151

[ref50] Whitham M. Parker B. L. Friedrichsen M. Hingst J. R. Hjorth M. Hughes W. E.. (2018). Extracellular vesicles provide a means for tissue crosstalk during exercise. Cell Metab. 27, 237.e4–251.e4. 10.1016/j.cmet.2017.12.001, PMID: 29320704

[ref51] WHO (2011). Pulse oximetry training manual. Available at: https://www.who.int/patientsafety/safesurgery/pulse_oximetry/who_ps_pulse_oxymetry_training_manual_en.pdf?ua=1 (Accessed September 10, 2019).

[ref52] Wu T. Kayser B. (2006). High altitude adaptation in Tibetans. High Alt. Med. Biol. 7, 193–208. 10.1089/ham.2006.7.193, PMID: 16978132

[ref53] Yoshiko A. Katayama K. Ishida K. Ando R. Koike T. Oshida Y.. (2020). Muscle deoxygenation and neuromuscular activation in synergistic muscles during intermittent exercise under hypoxic conditions. Sci. Rep. 10:295. 10.1038/s41598-019-57099-y, PMID: 31941906 PMC6962371

